# Women’s cellphone access and ownership in rural Uganda: implications for self-care interventions

**DOI:** 10.1186/s44263-024-00038-5

**Published:** 2024-02-05

**Authors:** Willow Leahy, Maryam Abomoslim, Amy Booth, Anna Gottschlich, Nelly Mwandacha, Hallie Dau, Priscilla Naguti, Beth Payne, Laurie Smith, Carolyn Nakisige, Gina Ogilvie

**Affiliations:** 1 Queen’s University, Kingston, Canada; 2 Women’s Health Research Institute, Vancouver, Canada; 3 School of Population and Public Health, UBC, Vancouver, Canada; 4 Karmanos Cancer Institute, Wayne State University, Detroit, USA; 5 Uganda Cancer Institute, Kampala, Uganda; 6 BC Cancer, Vancouver, Canada; 7 BC Centre for Disease Control, Vancouver, Canada

**Keywords:** Human papillomavirus, Cervical cancer, Human papillomavirus DNA tests, Self-care interventions, Mobile health, Global health, Developing countries, Low-resource setting, Self-collection, Cervical cancer screening

## Abstract

**Background:**

The World Health Organization (WHO) call for cervical cancer elimination includes increasing global cervical screening coverage. HPV-based self-collection (HPV-SC) is a promising screening model for low- and middle-income countries (LMICs), and while digital technology, such as cellphones, can be used to streamline HPV-SC, there is limited data on digital technology penetration in LMICs. Determining women’s cellphone access is critical to understanding the feasibility of using cellphones to support HPV-SC.

**Methods:**

This study is a secondary analysis of a larger clinical trial. Participants of a cluster-randomized trial comparing HPV-SC models in Uganda completed a survey, including questions about demographics, cellphone access/ownership, prior cervical cancer screening (CCS), and willingness to receive CCS information by text. A logistic regression model was used to determine adjusted rates of cellphone ownership using survey variables as factors.

**Results:**

Of 2019 participants, 76.1% owned a cellphone. In non-cellphone owners (*n* = 483), 82.4% had daily cellphone access and 7.3% had no access. Compared to non-cellphone owners, cellphone owners were significantly older, more educated, closer to major health centers, more likely to have prior CCS, and more willing to receive a CCS text. In the logistic regression model, the aforementioned variables were all significantly associated with the odds of owning a cellphone.

**Conclusions:**

As health care systems consider adopting HPV-SC, it is imperative to understand digital technology penetration. The majority of participants were cellphone owners and were willing to receive CCS information by text; however, significant socioeconomic and demographic differences remain between cellphone owners and non-owners. Further investigation is needed to understand whether HPV-SC using cellphones is feasible in similar settings.

**Trial registration:**

ISRCTN, 12767014. ClinicalTrials.gov, NCT04000503.

**Supplementary Information:**

The online version contains supplementary material available at 10.1186/s44263-024-00038-5.

## Background

Access to essential health services remains a critical barrier to achieving universal health care; approximately 3.6 billion people globally lack access to health care, particularly in low- and middle-income countries (LMICs). To address this gap, the World Health Organization (WHO) has endorsed self-care to promote health and improve health care coverage in all settings [1]. Self-care is where individuals are ‘active agents’ in managing their own health care, with or without the supervision of health workers [2]. Self-care interventions include a wide range of evidence-based interventions including medicines, diagnostics, and digital technologies, all with the aim of empowering individuals and communities to manage and optimize their health and well-being, while supporting the efficient use of scarce health resources.

Self-care has the potential to achieve many specific health goals in LMICs, including the WHO’s global goal to eliminate cervical cancer [2]. While largely considered a curable and avoidable cancer, the majority of cervical cancer cases and deaths each year are in LMICs [3] due to low vaccine and screening rates. Compared to traditional screening methods, HPV-based cervical cancer screening has been found to be more sensitive and less costly and has the capacity to be done using self-collected vaginal samples while remaining reliable [4, 5]. Moreover, self-collection for cervical screening as a form of self-care has been shown to be highly acceptable and feasible in high-burden, low-resource settings [6], but there are still opportunities for improvements in screening coverage and follow-up attendance. The uptake of cervical cancer screening is lowest in rural Uganda [7]. Further, in a recent Ugandan pragmatic cluster-randomized trial comparing two HPV-based self-collection models, attendance at follow-up after completing HPV-based self-collection was approximately 75% [8], highlighting the need for innovative strategies to increase both screening uptake and follow-up adherence. Self-collection screening offers a patient-centered, autonomy-granting form of self-care, and integrating digital health solutions can enhance the movement towards cervical cancer screening programs that are rooted in self-care.

Reliable access to digital technology, particularly personal cellular phones, is often a critical requirement for effective self-care practices and interventions [1]. Mobile phones have been identified as a potential tool to deliver health information and services [9–11]. Digital health technologies, specifically Mobile Health or mHealth, could be used in cervical screening at many stages, including facilitating invitations for screening, dissemination of screening results, and recommendations for the next steps in care such as treatment appointments and health education. Introducing digital health technologies into the cervical screening pathway may increase screening coverage and follow-up attendance.

However, in LMICs, where access to health care is lowest [12] and disease burden is disproportionally highest [13], there is limited data on cellphone penetration and digital literacy. A report from the UN Capital Development Fund found that in 2019, 69% of Ugandan women owned a cellphone [14]. While more recent Uganda-specific data is unavailable, a 2021 report found that 83% of women in LMICs own a cellphone in line with an upward global trend of mobile phone ownership amongst women [15]. While mobile phone usage in Uganda has increased dramatically within the past two decades, with 71% of Ugandans reportedly owning a mobile phone [16], there is a lack of statistics amongst those at risk of cervical cancer: adult women with limited access to health care services. As health care systems move towards implementing digital health-based self-care interventions, it is essential to understand access to the digital technologies that enable self-care, particularly for women who, in LMICs and globally, often have less economic and social autonomy [17–19], and thus may have less access to or control of digital technology [20]. Although they may intend to reduce inequity, it is crucial to ensure that digital-based interventions do not have the unintentional consequence of worsening inequity due to disproportionate access to digital tools.

In this study, we examine women’s cellphone ownership and access in a rural LMIC setting in Uganda and examine the implications of cellphone access for self-care health programs that rely on digital technology.

## Methods

### Study design and setting

This study is a secondary analysis of the ASPIRE Mayuge trial, registration #NCT04000503. The protocol and primary outcomes have previously been published [21–23]. Advances in Screening and Prevention in Reproductive Cancers (ASPIRE)-Mayuge was a pragmatic, sequential, two-arm cluster randomized trial conducted in the Mayuge district of eastern Uganda between August 2019 and July 2021. ASPIRE-Mayuge compared attendance at follow-up after either door-to-door recruitment by village health teams (VHTs) or community health days for HPV-based cervix screening. Mayuge district is divided into 3 major geographical settings: mainland, islands, and the forest reserve. This research was conducted in the mainland setting of Mayuge, considered to be the “urban” part of the district. As part of recruitment, women completed a cross-sectional survey at baseline and answered questions about cellphone ownership, cellphone access, and various other health and demographic variables. The survey was administered orally by the village health worker in English or Lusoga, dependent on participant preference, and participants were compensated in local currency for their time and travel costs.

### Study eligibility

Women living in the Mayuge district in one of the 31 study villages who were between the ages of 25 and 49 years of age and had no previous history of cervical cancer, cervical pre-cancer, or hysterectomy met the inclusion criteria and were invited to participate in the trial. Women who were outside of the specified age range, who previously had a hysterectomy, or who had previously been screened or treated for cervical cancer were excluded from eligibility. All trial participants were given the opportunity to complete the cross-sectional survey, where participation in the survey was required for their participation in the screening program.

### Measurements

The survey included a total of 36 questions, including questions with skip and branching logic (Additional file 1). The survey included demographics as well as items on access to cellphones and attitudes towards screening and care. Descriptive statistics collected in the survey, including demographic and screening history, were summarized. To capture mobile phone access as an exposure, women were asked if they had access to a mobile phone. Participants who answered “yes” were grouped into the category of “Cellphone access” and those who answered “no” were categorized as “No cellphone access.” Both “no” and “don’t know” options were collapsed together and designated as not having access to a mobile phone. To capture mobile phone ownership, participants who answered “yes” were further categorized into “Owns cellphone” and “Does not own cellphone” based on the response to the follow-up question: “Who owns the phone you have access to?”.

The following variables were compared between cellphone owners and non-owners, as well as those with cellphone access and those without, to determine if there was a significant difference between groups: age, marital status, education level, number of pregnancies, if they visited a health center (HC) in the last 12 months, distance from HC, if they have previously been screened for cervical cancer, willingness to receive cervical cancer screening (CCS) at a HC, and willingness to receive CCS information via SMS. Distance from a health center was considered both as mean number of minutes and as a categorical variable broken down into 5 groups: < 30 min, 30–59 min, 60–89 min, 90–120 min, and > 120 min.

### Analysis

Analysis was conducted utilizing R version 4.3.0 [24] and R Studio [25]. A bivariate statistical analysis was conducted to compare the outcomes of those with mobile phone access with those without mobile phone access, and those who own phones with those who do not. We performed independent two-sample *t*-tests for continuous variables and chi-square tests for most categorical variables, except when there were low frequencies in which case a Fisher’s exact test was used. Both unadjusted and adjusted odds ratios were calculated using a logistic regression model to investigate the odds of cellphone ownership and cellphone access given the covariate(s). Regression models were adjusted for all independent covariates (Table 4) and the covariates were selected a priori. *P* values less than 0.05 were considered statistically significant.

## Results

In total, 2019 participants completed the survey; the mean age of participants was 34 years, the majority of participants were in a relationship (85.4%, *n* = 1724), most had primary education or lower (67.0%, *n* = 1353), and the mean number of pregnancies was 5.61 (Table 1).

**Table 1:** Participant characteristics

Variables	Options	Total (*N* = 2019)
Age	Mean	34.25
	Range	25–49
	Standard deviation	7.62
Marital status	Married	1724 (85.4%)
	Divorced	78 (3.9%)
	Single	179 (8.9%)
	Widow	34 (1.7%)
Education	None	262 (13%)
	Primary	1091 (54%)
	O level	562 (27.8%)
	A level	26 (1.3%)
	Tertiary	77 (3.8%)
Number of pregnancies	Mean	5.61
*(How many times have you been pregnant?)*	Range	0–19
	Standard deviation	5.06
Visited health center in the last 12 months	Yes	1631 (80.8%)
Distance (min) from HCIII	Mean	49.07 min
	Range	1–360
	SD	41.08
Distance (min) from HCIII	< 30min	560 (27.7%)
	30–59min	507 (25.1%)
	60–89min	672 (33.3%)
	90–119min	33 (1.6%)
	> 120	245 (12.1%)
Previously tested by healthcare worker for CC	Yes	44 (2.2%)
*(Has a healthcare worker ever tested you for CC?)*	No	1975 (97.8%)
Willing to receive integrated CC	Yes	1995 (98.8%)
*(Would you be interested in receiving CCS when you go to HC?)*	No	21 (1%)
	*Missing*	*3 (0.1%)*
Willing to receive CCS info text	Yes	1918 (95%)
*(Would you be interested in receiving SMS with more info on ccs when it is available at HC?)*	No	92 (4.6%)
	Missing	9 (0.4%)

*HC* health center, *CC* cervical cancer, *CCS* cervical cancer screening, *SMS* short messaging service

In the previous 12 months, 80.8% (*n* = 1631) of participants had visited a health center, and the average distance to a health center was 49.07 min. Only 2.2% (*n* = 44) of participants reported a prior CCS, while 98.8% (*n* = 1995) indicated their willingness to receive CCS during a health center visit and 95.0% (*n* = 1918) indicated their willingness to receive information about CCS via SMS in the future.

**Fig. 1:**
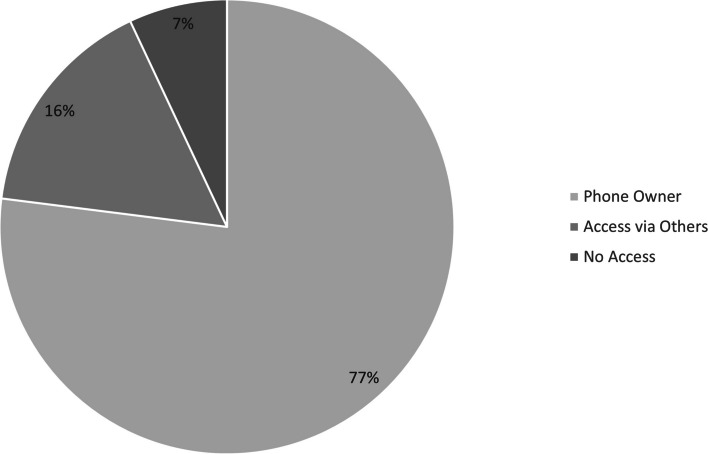
Distribution of women (participants) who are cellphone owners versus those who have cellphone access through family or a neighbor versus those with no cellphone access

Of 2019 participants surveyed, 76.1% (*n* = 1536) owned a cellphone and 23.9% (*n* = 483) did not own a cellphone. Of the 483 who did not own a cellphone, 14.5% had access through a family member who owned a phone, 2.0% had access through a neighbor who owned a phone, and 0.1% had access through other means. Overall, access to a cellphone, through personal ownership or via others, was reported by 92.7% (*n* = 1872) of participants. Only 7.3% (*n* = 147) of all participants reported not having access to a phone (Fig. 1).

When comparing cellphone owners (*n* = 1536) and non-cellphone owners (*n* = 483), there was a significant difference in age (34.5 vs 33.6, *p* < 0.01), education (*p* < 0.01), number of pregnancies (5 vs 6, *p* < 0.01), distance to level III HC (45.3 min vs 67.4 min, *p* < 0.01), and prior CCS (*p* = 0.012) (Table 2).

**Table 2:** Demographic breakdown by cellphone ownership

*Variables*	Options	Phone owner (*N* = 1536)		Non-owner (*N* = 483)		*p*-value
**Age**	*Mean*	34.47		33.55		*p*-value < 0.001
	*Range*	25–49		25–49		
	*SD*	7.61		7.60		
**Marital status**	*Married*	1297	84.4%	427	88.4%	*p*-value = 0.06
	*Divorced*	61	4.0%	17	3.5%	
	*Single*	150	9.8%	29	6.0%	
	*Widow*	24	1.6%	10	2.1%	
**Education**	*None*	152	9.9%	110	22.8%	*p*-value < 0.001
	*Primary*	791	51.5%	300	62.1%	
	*O level*	490	31.9%	72	14.9%	
	*A level*	25	1.6%	1	0.2%	
	*Tertiary*	77	5.0%	0	0.0%	
**Number of pregnancies**	*Mean*	5.48		6.00		*p*-value < 0.001
	*Range*	0–19		0–16		
	*SD*	3.01		3.18		
**Visited health center** *(Have you visited a HC in the last 12 months)*	*Yes*	1251	81.4%	380	78.7%	*p*-value = 0.22
	*No*	284	18.5%	102	21.1%	
**Distance (min) from HCIII**	*Mean*	45.30 min		61.07 min		*p*-value < 0.001
	*Range*	1–360 min		1–300 min		
	*SD*	39.56		43.51		
**Distance (min) from HCIII**	< *30 min*	464	30.2%	96	19.9%	*p*-value < 0.001
	*30*–*59 min*	430	28.0%	77	15.9%	
	*60*–*89 min*	468	30.5%	204	42.2%	
	*90*–*119 min*	24	1.6%	9	1.9%	
	> *120*	149	9.7%	96	19.9%	
**Prior CCS** *(Has a healthcare worker ever tested you for CC?)*	*Yes*	41	2.7%	3	0.6%	*p*-value = 0.01
	*No*	1495	97.3%	480	99.4%	
**Willing to CCS integration** *(Would you be interested in receiving CCS when you go to HC?)*	*Yes*	1520	99.0%	475	98.3%	*p*-value = 0.20
	*No*	13	0.8%	8	1.7%	
	*Missing*					
**Willing to receive CCS info text**	*Yes*	1503	97.9%	415	85.9%	*p*-value < 0.001
	*No*	29	1.9%	63	13.0%	

*HC* health center, *CC* cervical cancer, *CCS* cervical cancer screening

When asked specifically if they would be interested in receiving an SMS with more information on cervical cancer screening when it is available at HC, 98% of phone owners compared to 86% of non-owners responded yes (*p* < 0.01). Relatedly, there was a significant difference in age (34.3 vs 34.0, *p* < 0.01), education (*p* < 0.01), number of pregnancies (*p* < 0.01), and distance to Level III HC (48 min vs 61 min, *p* < 0.01), as well as prior HC visit (82% vs 67%, *p* < 0.01) between those with cellphone access (*n* = 1872) and those without (*n* = 147) (Table 3).

**Table 3:** Demographic breakdown by cellphone access

*Variables*	Options	Phone access (*N* = 1872)		No access (*N* = 147)		*p*-value
**Age**	*Mean*	34.27		34.05		*p*-value < 0.001
	*Range*	25–49		25–49		
	*SD*	7.64		7.32		
**Marital status**	*Married*	1594	85.1%	130	88.4%	*p*-value = 0.10
	*Divorced*	73	3.9%	5	3.4%	
	*Single*	172	9.2%	7	4.8%	
	*Widow*	29	1.5%	5	3.4%	
**Education**	*None*	217	11.6%	0	0.0%	*p*-value < 0.001
	*Primary*	1004	53.6%	45	30.6%	
	*O level*	547	29.2%	87	59.2%	
	*A level*	26	1.4%	15	10.2%	
	*Tertiary*	77	4.1%	0	0.0%	
**Number of pregnancies**	*Mean*	5.56		6.22		*p*-value < 0.001
	*Range*	0–19		0–16		
	*SD*	3.05		3.13		
**Visited health center** *(Have you visited a HC in the last 12 months)*	*Yes*	1532	81.8%	99	67.3%	*p*-value < 0.001
	*No*	339	18.1%	47	32.0%	
**Distance (min) from HCIII**	*Mean*	47.63 min		67.49 min		*p*-value < 0.001
	*Range*	1–360 min		1–300 min		
	*SD*	40.48		44.32		
**Distance (min) from HCIII**	< *30 min*	538	28.7%	22	15.0%	*p*-value < 0.001
	*30–59 min*	486	26.0%	21	14.3%	
	*60–89 min*	609	32.5%	63	42.9%	
	*90–119 min*	30	1.6%	3	2.0%	
	> *120*	207	11.1%	38	25.9%	
**Prior CCS** *(Has a healthcare worker ever tested you for CC?)*	*Yes*	44	2.4%	0	0.0%	*p*-value = 0.11
	*No*	1828	97.6%	147	100.0%	
**Willing to CCS integration** *(Would you be interested in receiving CCS when you go to HC?)*	*Yes*	1850	98.8%	145	98.6%	*p-*value = 0.66
	*No*	19	1.0%	2	1.4%	
	*missing*					
**Willing to receive CCS info text**	*Yes*	1811	96.7%	107	72.8%	*p*-value < 0.001
	*No*	56	3.0%	36	24.5%	
	*Missing*					

*HC* health center, *CC* cervical cancer, *CCS* cervical cancer screening

Similarly, when asked about interest in receiving an informational SMS on cervical cancer screening, 97% of women with phone access responded yes (*p* < 0.01). Amongst women who have no cellphone access at all, surprisingly, 73% indicated that they would be interested in receiving information on cervical cancer screening via SMS if possible. Notably, there was a resoundingly positive response to inquiries about receiving cervical cancer screening during a health center visit across all access levels (99%).

In the adjusted logistic regression model investigating cellphone ownership (Table 4), the following factors were found to be significantly associated with higher odds of owning a cellphone: higher age, higher attained education, fewer number of pregnancies, shorter distance to Level III HC, prior cervical cancer screening, willingness to receive CCS info by text.

**Table 4:** Logistic regression results by cellphone ownership

Variable^†^	UOR	95% CI	AOR	95% CI
Age (mean)	1.02*	1.00–1.03	1.06	1.04–1.08
Marital status	1.16	0.99–1.37	1.07	0.90–1.28
Education	2.35***	2.01–2.76	2.49	2.08–3.00
# of pregnancies	0.95**	0.92–0.98	0.95	0.91–1.00
HC visit	1.18	0.92–1.52	1.06	0.80–1.40
HC distance categorical	0.72***	0.66–0.78	0.69	0.62–0.78
CP screened	4.39*	1.59–18.18	3.66	1.17–16.53
Integrate CCS HC	1.97	0.78–4.70	1.23	0.04–3.33
Integrate CCS Text	7.87***	5.05–12.54	9.25	5.62–15.65

All models include a random intercept

*AOR* adjusted OR, *CCS* cervical cancer screening, *HC* health center, *CP* prior cervix screening, *Int* integrate

^†^Adjusted models are adjusted for all independent variables

Significant levels: *** = 0, ** = 0.001, * = 0.01

In the adjusted logistic regression model investigating cellphone access (Table 5), the following factors were found to be significantly associated with higher odds of having access to a cellphone, regardless of ownership: higher age, higher attained education, HC visit in previous 12 months, shorter distance to Level III HC, willingness to receive CCS info text.

**Table 5:** Logistic regression results by cellphone access

Variable^†^	UOR	95% CI	AOR	95% CI
Age (mean)	1.00	0.98–1.03	1.04	1.01–1.78
Marital status	1.09	0.85–1.44	1.00	0.76–1.35
Education	2.73***	2.11–3.57	2.92	2.14–4.04
# of pregnancies	0.93*	0.89–0.99	0.98	0.91–1.06
HC visit	2.15***	1.48–3.08	2.17	1.43–3.24
HC distance categorical	0.67***	0.59–0.76	0.66	0.57–0.76
Integrate CCS HC	1.34	0.21–4.69	0.51	0.07–2.24
Integrate CCS text	10.88***	6.82–17.22	11.46	6.80–19.27

All models include a random intercept

*AOR*, adjusted OR; *CCS*, cervical cancer screening; *HC*, health center; *Cat*, categorical; *Int*, integrate

^†^Adjusted models are adjusted for all independent variables

Significant levels: *** = 0, ** = 0.001, * = 0.01

## Discussion

This cross-sectional analysis utilized survey data collected as part of the larger ASPIRE Mayuge cervical cancer screening study [21–23]. The purpose of this sub-study was to investigate cellphone ownership and cellphone access in rural Uganda and to consider the implications for self-care, with a particular focus on cervical screening and cancer prevention. More than three-quarters (76.1%) of women surveyed owned their cellphone. The population surveyed is representative of other semiurban areas in LMICs and suggests that even in low-resource, remote settings, there may be high penetration of cellphones, making self-care with digital technology a potentially viable option.

More importantly, while attendance at a health center was high across all categories, women who owned or had access to a mobile phone lived closer to a health center compared to the no access group, suggesting a negative correlation between rurality and cellphone ownership. Regardless of access, the vast majority of women were receptive to the offer of receiving information about cervical screening via SMS if available, which suggests the feasibility of using digital technology to support cervical cancer prevention initiatives. This is consistent with the literature where, in a 2019 cluster-randomized trial, Huchko et al. found that women in rural Kenya preferred the use of mobile phone results notification over home visit results notification for cervical cancer screening [26]. Similarly, in a 2022 inquiry into the acceptability of text messages for cervical cancer screening in Tanzania, Lokke et al. observed support for educative and reminder text messages [27]. In a 2020 analysis of health outcomes from 15 countries including Uganda, LeFevre et al. found mobile phone ownership to be associated with increased odds of attending antenatal care clinic visits [28]. Initiatives that improve smartphone access in more rural areas, such as the Ministry of Information and Communication Technology and Innovation (MINICT)’s ConnectRwanda Initiative, can significantly bridge urban–rural gaps in access [29]. While mobile phone ownership may be a factor that facilitates access to services, we recognize that other factors may be confounding its impact on health service seeking.

Over 7% of the surveyed women did not own a cellphone, nor had cellphone access. These women are likely the lowest SES group, as indicated by lower education, more total pregnancies, and further distance to health centers. Attention needs to be given to this subpopulation in particular when considering the adoption and implementation of self-care via digital technologies to avoid exacerbating any existing inequities. Rwanda’s recognition of the potential for digital health technologies in health care has led to initiative ensuring all citizens are either provided a cellphone or able to access digital health self-care intervention through a village-appointed health worker. There must be similar provisions put in place in Uganda, at the health care system level, to ensure care is still accessible for those without the resources to participate in programs where self-care is centered around digital technology.

About 17% of women surveyed had access to a cellphone that they did not own. It requires further investigation of women who lacked direct ownership of a cellphone, emphasizing the need to explore the dynamics and potential barriers associated with shared phone access, especially concerning health-related communications. In a Kenyan study, 55.2% of cellphone sharers felt comfortable receiving screening results via SMS versus 70.7% of cellphone owners, and those who did not own their cellphones were less likely to attend treatment [30]. These findings indicate that there are added barriers to the access group compared to the ownership group, in terms of attendance to follow-up. Unfortunately, the literature on the differences between cellphone access versus ownership in relation to self-care is limited and poorly defined.

Despite the willingness to receive information on cervical cancer screening via text, most women in all groups had limited to no prior cervical cancer screening. This finding was also reported in a community-based cluster survey conducted by Twinomujuni et al. in 2015, where a low participation in screening was found despite a high intention to screen amongst women surveyed [31]. While women are responding favorably to testing and education on cervical cancer outcomes, these attitudes are not translating into practice towards screening. The low screening rates found in our analysis and the work conducted by Twinomujuni et al. are consistent with screening patterns in sub-Saharan Africa. Studies have found participation in cervical cancer screening to be 12.87% across sub-Saharan Africa, with a study in Eastern Uganda reporting a 4.8% screening rate [7, 32]. This demonstrates that other factors continue to impact the uptake of cervical cancer preventative services and suggest a need for further inquiry into interventions that address cervical cancer accessibility and availability issues in rural Uganda. Given the limited availability of healthcare resources in rural regions such as Malongo, future research could probe the potential impacts of mHealth interventions on screening uptake given the largely positive view of prevention in the region. By continuing inquiries that enable us to harness mobile technology to facilitate the uptake of screening and prevention measures, continuous progress towards the elimination of cervical cancer will be realized, both in Uganda and globally.

### Strengths and limitations

This research is strengthened by employing VHTs to administer the survey; this is shown to improve community partnerships, sustainability, and cultural sensitivity as participants deliver responses in their native language to a known and trusted member of the community [33]. However, the use of survey research is a limitation as it requires respondents to self-report responses. In addition, the survey was administered verbally by a VHT. Consequently, it is possible that recall bias or social desirability bias resulted in the misrepresentation of attitudes, knowledge, and experiences [34]. Further, the study population was recruited exclusively from Mayuge county and may not be generalizable across other LMIC regions. Finally, we had limited knowledge of the extent of mobile phone utilization by the women surveyed. As such, the assumption that mobile phone access translated to mobile phone use had to be made throughout the analysis. Further investigation into household income, type of phone (basic/non-internet phone versus smartphone), usage patterns, digital literacy, availability of electricity, and mobile credit for cellphone usage and upkeep, as well as frequency of use, is critical for future analyses.

## Conclusions

This study provides insights into the intersection of cellphone access, self-care, and cervical cancer prevention. The findings of this study underscore the significant potential of self-care interventions facilitated by digital technology, particularly in the context of women in rural and remote settings like Mayuge, Uganda. The high ownership and access to cellphones amongst the surveyed women coupled with positive reception towards receiving such information via mobile phones suggests a promising avenue to leverage digital health. It is important to note that the observed association between cellphone ownership/access and factors such as age, education level, proximity to health centers, and prior screening history illuminates disparities in technology access across different demographic groups. Despite the willingness expressed by participants, the low rates of prior cervical cancer screening persist, suggesting underlying barriers beyond technology access. Addressing these barriers, such as limited healthcare resources, access issues, and broader socio-cultural challenges, requires multifaceted interventions that extend beyond digital technology. A comprehensive understanding of technology use alongside targeted interventions is imperative for leveraging digital health solutions to bridge gaps in healthcare access without exacerbating existing inequalities.

## Supplementary Information

**Additional file 1.** Baseline Survey. Complete baseline survey administered to trial participants.

## Data Availability

Data access for the ASPIRE Mayuge trial, restricted to non-identifying data owing to privacy concerns, can be requested only for scientific purposes from the corresponding or senior authors, who will handle all requests. Either data will be shared through an institutional data-sharing agreement or arrangements will be made for analyses to be conducted remotely without the necessity for data transfer.

## Abbreviations

ASPIRE: Advances in Screening and Prevention in Reproductive Cancers
CCS: Cervical cancer screening
HC: Health center
LMIC: Low- and middle-income countries
mHealth: Mobile health
MINICT: Ministry of Information and Communication Technology and Innovation
VHT: Village Health Team
WHO: World Health Organization
