# Supplementary material for: Women’s cellphone access and ownership in rural Uganda: implications for self-care interventions
Source: BMC Glob Public Health. 2024 Feb 5;2:8. doi: 10.1186/s44263-024-00038-5 (PMC11622906; doi:10.1186/s44263-024-00038-5)
Supplement: Supplementary file 1 — Additional file 1. Baseline Survey. Complete baseline survey administered to trial participants. [file 44263_2024_38_MOESM1_ESM.docx]

INSTRUCTIONS: This form should be completed for each woman who provides verbal consent.

1. The first column includes the questions to ask each woman. Clarification of how to ask the question or fill in the response are provided in brackets () after questions when needed.
2. Write the woman’s answer for each question in the second column or box beside each question.
3. For yes/no questions, do not ask her if don’t know is her response. Don’t know should only be chosen if she says she cannot answer yes or no to the question.
4. For questions that have a list of possible responses in the answer section, ask the woman the question first and then review the listed options with her and **circle** her chosen response.
5. Never leave a question blank. If a woman refuses to answer a question mark an **X** for the answer. If she doesn’t know how to answer write **UNKNOWN** or **UNK** unless Don’t know has been provided as an option.

**Section 1: Socio-demographic variables**

| **Q** | **Question**  ***(Instructions provided below each question)*** | **Answer**  ***(write in the response or circle the option the woman selects from the list provided)*** | | | |
| --- | --- | --- | --- | --- | --- |
| 1 | How old are you?  *(Provide a number or best estimate of age)* |  | | | |
| 2 | What is your marital status? | Married  Separated/ Divorced  Single  Widowed | | | |
| 3 | What is the highest level of education you attained? | None  Primary (P1 to P7)  O level (S1 to S4)  A level (S5 to S6)  Tertiary education/University | | | |
| 4 | What is the highest level of education attained by your partner? | None  Primary (P1 to P7)  O level (S1 to S4)  A level (S5 to S6)  Tertiary education/University | | | |
| 5 | Do you have access to a mobile phone?  *(Clarify this could be her own or through friends or neighbors)* | Yes *(continue survey)*  No *(skip to Q8)*  Don’t know *(skip to Q8)* | | | |
| 6 | If yes, who owns the phone you have access to? | Own Phone  Household Member  Neighbour  Other:_________________ | | | |
| 7 | If yes, how frequently do you have access to this phone? | Daily  2-3times/ week  Weekly  Monthly  Seldom  Don’t know | | | |
| 8 | How many times have you been pregnant in your life?  *(Provide a number including all pregnancies even those that were lost early)* | *(if the answer is 0 skip to Q11)* | | | |
| 9 | How old were you the first time you gave birth?  *(Provide number for age)* |  | | | |
| 10 | How many living children do you have?  *(This includes all of the children the woman gave birth to who are alive today)* |  | | | |
| 11 | Have you ever been diagnosed with any of the following conditions?  *(Circle any conditions the woman knows she has now or has had in the past)* | HIV  TB  Malaria Syphilis Chlamydia  Gonorrhea Don’t know | | | |
| 12 | Have you visited a Health Centre in the last 12 months?  *(Clarify - This could include going to a Health Centre for her own illness or someone else’s and includes any Health Centre level 2-4, give an example of Kigandalo HCIV or Buwaisawa HCIII)* | Yes *(continue survey)*  No *(skip to Q15)*  Don’t know *(skip to Q15)* | | | |
| 13 | Did you attend for yourself or as a caregiver for someone else?  *(Clarify you are asking if she attended the health center for her own (self) illness or her child’s illness (caregiver) or because another friend or family member was sick)* | Self  Caregiver (for child)  For other person  All of the above | | | |
| 14 | If yes, Which health center and why?  *(Write the name of each health center she went to)* | Health Centre name (1): __________________________  Health Centre name (2): __________________________  Health Centre name (3): __________________________ | | | |
|  | *(Mark an X for the reason she went to the corresponding number of the health center listed above. If the reason isn’t listed write it under “other” reasons)* | 1. Child Health concern 2. Family planning 3. Other reproductive health concern 4. ANC 5. Diabetes 6. Hypertension 7. Acute Injury or Accident 8. HIV/ARV 9. Malaria (flu, fever) 10. Tuberculosis 11. Other reasons _____________­­ _____________ | 1 | 2 | 3 |
| 15 | How many minutes does it take to get to the nearest health center from your home? |  | | | |

**Section 2: CORE PLUS module of the IDCCP survey (1)**

Please read out the following: *“Now I’m going to ask you about tests a health-care worker can do to check for cervical cancer. The tests a health-care worker can do to check for cervical cancer are called a Pap smear, HPV test, and VIA test.”*

*If she is uncertain of these terms you can read the following statements to clarify:*

***Pap smear supplementary statement:*** *“For a Pap smear test, a health-care worker puts a small stick or swab inside the vagina to wipe the cervix, and sends the sample to the laboratory.” (Optional: show reference images here)*

***HPV test supplementary statement:*** *“For an HPV test, a small stick or swab is put inside the vagina to wipe the cervix, and the sample is sent to the laboratory. This can be done by a health-care provider or by a woman herself.” (Optional: show reference images here)*

***VIA supplementary statement:*** *“For a VIA test, a health-care worker puts vinegar on the cervix and looks to see if the cervix changes colour.” (Optional: show reference images here)*

| **Q** | **Question**  ***(Instructions provided below each question)*** | **Answer**  ***(write in the response or circle the option the woman selects from the list provided)*** |
| --- | --- | --- |
| 16 | Have you heard of cervical cancer before?  *If necessary, clarify terms by reading the following: “The uterus is where a baby grows when a woman is pregnant. The cervix connects the uterus to the vagina.” (Optional: show image of cervix here)* | Yes  No  Don’t know |
| 17 | Has a healthcare worker ever tested you for cervical cancer? | Yes *(continue survey)*  No *(skip to Q27)*  Don’t know *(skip to Q27)* |
| 18 | At what age where you first tested for cervical cancer? |  |
| 19 | When was your last test for cervical cancer? | Less than 1 year ago  1-2 years ago  3-5 years ago  More than 5 years ago  Don’t know |
| 20 | What is the MAIN reason you had your last test for cervical cancer?  *(select only one reason)* | Part of routine examination  Follow up on abnormal or inconclusive result  Recommended by health-care provider  Recommended by other source  Experiencing pain or other symptoms  Other (specify): ______________________  Don’t know |
| 21 | Where did you receive your last test for cervical cancer? | Doctors office  Mobile clinic  Community clinic  Hospital  Other (specify): ______________________  Don’t know |
| 22 | What was the result of your last test for cervical cancer? | Did not receive result *(end survey here)*  Normal/negative *(continue survey)*  Abnormal/positive *(continue survey)*  Suspect cancer *(continue survey)*  Inconclusive *(continue survey)*  Don’t know *(continue survey)* |
| 23 | Did you have any follow-up visits because of your test results? | Yes  No  Don’t know |
| 24 | Did you receive any treatment to your cervix because of your test result? | Yes *(continue survey)*  No *(skip to Q26)*  Don’t know *(end survey here)* |
| 25 | Did you receive the treatment to your cervix during the same visit as your last test for cervical cancer?  *(Clarify this means she was treated on the same day as her test)* | Yes *(end survey here)*  No *(continue survey)*  Don’t know *(end survey here)* |
| 26 | What is the MAIN reason you did not receive treatment?  *(select only one reason)*  *(Any woman who has had a cervical cancer screening test should END SURVEY after this question)* | Was not told I needed treatment  Did not know how/where to get treatment Embarrassment  Too expensive  Didn’t have time  Clinic too far away  Poor service quality  Afraid of the procedure  Afraid of social stigma  Cultural beliefs  Family member would not allow it (specify the relationship of the member to the respondent) ______________________  Other (specify): ______________________  Don’t Know |
| 27 | What is the MAIN reason you have never had a cervical cancer screening test?  *(select only one reason)* | Did not know how/where to get the test  Embarrassment  Too expensive  Didn’t have time  Clinic too far away  Poor service quality  Afraid of the procedure  Afraid of social stigma  Cultural beliefs  Family member would not allow it (specify the relationship of the member to the respondent) ________________  Other (specify): _______________  Don’t Know |

**Section 3: PREFERENCES ON INTEGRATED SERVICE DELIVERY**

Please read out the following statement prior to beginning this section: *“I am going to ask you some questions about your preferences on how you receive health services. When I ask you questions about cervical cancer screening, I am asking about a cotton bud that you or a health care provider would insert in your vagina to check for cervical cancer. It is a painless test. This test can be done by you or by your health provider and would only take a few minutes.”*

| 28 | When you go to a health centre, do you usually access more than one service on the same day for you or your family members?  *(clarify that this means the woman may go with her child or other family member to both get care at the same time but not necessarily at the same clinic/ facility)* | Yes  No  Don’t know |
| --- | --- | --- |
| 29 | What are the health services you and your family members most commonly access?  *(circle all that apply)* | Child Health  Sexual/Reproductive Health  ANC  HIV/ARV  Chronic Condition (Diabetes, Hypertension, etc)  Acute care (flu, fever)  Malaria  Tuberculosis  Other _______________________________ |
| 30 | Would you be interested in receiving cervical cancer screening (swab) when you go to the health centre? | Yes *(continue survey)*  No *(skip to Q32)*  Don’t know *(skip to Q32)* |
| 31 | During which services would you be interested in receiving cervical cancer screening?  *(circle all that apply)* | Child Health  Sexual/Reproductive Health  ANC  HIV/ARV  Chronic Condition (Diabetes, Hypertension, etc)  Acute care (flu, fever)  Malaria  Tuberculosis  Other ________________________________ |
| 32 | Would you be interested in receiving cervical cancer screening (swab) while you are waiting for other health services? | Yes  No  Don’t know |
| 33 | What are your biggest challenges to accessing women’s health services? | Transportation  Lack of awareness of where to get services  Lack of awareness on what services I need  Not important/no time  Long wait times at facility  Low quality of care  Partner not supportive  Health care workers not receptive |
| 34 | Would you be interested in receiving a text message with more information on cervical cancer screening when it is available at the health centre? | Yes  No  Don’t know |

References

1. Improving Data for Decision-Making in Global Cervical Cancer Programs (IDCCP)

<https://www.who.int/ncds/surveillance/data-toolkit-for-cervical-cancer-prevention-control/en/>
